# Combination of chemically modified SDF‐1α mRNA and small skin improves wound healing in diabetic rats with full‐thickness skin defects

**DOI:** 10.1111/cpr.13318

**Published:** 2022-08-06

**Authors:** Zucheng Luo, Yujie Bian, Rui Zheng, Yonghuan Song, Li Shi, Haiting Xu, Huijing Wang, Xiaoyan Li, Zhenyu Tao, Anyuan Wang, Ke Liu, Wei Fu, Jixin Xue

**Affiliations:** ^1^ Department of Orthopaedics The Second Affiliated Hospital and Yuying Children's Hospital of Wenzhou Medical University Wenzhou China; ^2^ Zhejiang Provincial Key Laboratory of Orthopaedics Wenzhou China; ^3^ The Second School of Medicine Wenzhou Medical University Wenzhou China; ^4^ Department of Orthopaedics Hangzhou Fuyang Hospital of TCM Orthopedics and Traumatology Hangzhou China; ^5^ Department of Dermatology Shanghai Ninth People's Hospital, Shanghai Jiao Tong University School of Medicine Shanghai China; ^6^ Institute of Pediatric Translational Medicine, Shanghai Children's Medical Center, School of Medicine Shanghai Jiao Tong University Shanghai China; ^7^ Department of Pediatric Cardiothoracic Surgery, Shanghai Children's Medical Center, School of Medicine Shanghai Jiao Tong University Shanghai China; ^8^ Shanghai Key Laboratory of Tissue Engineering, Shanghai 9th People's Hospital, School of Medicine Shanghai Jiao Tong University Shanghai China

## Abstract

**Objectives:**

Diabetes mellitus is associated with refractory wound healing, yet current therapies are insufficient to accelerate the process of healing. Recent studies have indicated chemically modified mRNA (modRNA) as a promising therapeutic intervention. The present study aimed to explore the efficacy of small skin engineered to express modified mRNAs encoding the stromal cell‐derived factor‐1α (SDF‐1α) facilitating wound healing in a full‐thickness skin defect rat model. This study, devised therapeutic strategies for diabetic wounds by pre‐treating small skin with SDF‐1α modRNA.

**Materials and Methods:**

The in vitro transfection efficiency was evaluated using fluorescence microscopy and the content of SDF‐1α in the medium was determined using ELISA after the transfection of SDF‐1α into the small skin. To evaluate the effect of SDF‐1α modRNA and transplantation of the small skin cells on wound healing, an in vivo full‐thickness skin defect rat model was assessed.

**Results:**

The results revealed that a modRNA carrying SDF‐1α provided potent wound healing in the small skin lesions reducing reduced scar thickness and greater angiogenesis (CD31) in the subcutaneous layer. The SDF‐1α cytokines were significantly secreted by the small skin after transfection in vitro.

**Conclusions:**

This study demonstrated the benefits of employing small skin combined with SDF‐1α modRNA in enhancing wound healing in diabetic rats having full‐thickness skin defects.

## Graphical abstract

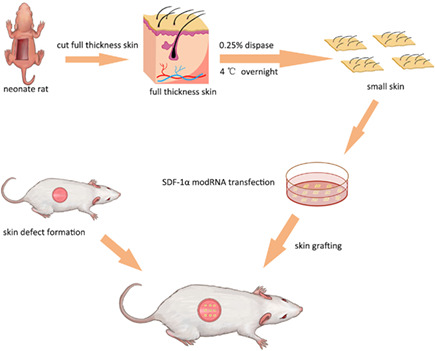

## 1 INTRODUCTION

Diabetes mellitus (DM) is a serious chronic metabolic condition posing significant clinical and public health problems worldwide. At present, about 463 million people have been diagnosed with diabetes worldwide, and the population is expected to reach 578 million by 2030 and 700 million by 2045.^1^ DM is associated with multiple complications, like diabetic nephropathy, diabetic retinopathy, and diabetic wounds. Chronic non‐healing diabetic wounds comprising the most serious complications associated with both type 1 and type 2 diabetes impose a severe clinical and economic burden on public health. Diabetic wounds are generally characterized by the lack of chemokine production, lack of angiogenesis, abnormal inflammatory responses, epithelization, and fibroblast dysfunction.^2^ For wound healing, a complex series of processes including haemostasis and coagulation, inflammation, proliferation, and wound regrowth with scarring are involved. The chemokine, stromal cell‐derived factor‐1 (SDF‐1α) is a C‐X‐C motif chemokine ligand 12 with several biological functions such as angiogenesis, inflammatory cell infiltration, and stem cell migration, which are critical for proper wound healing.^3^, ^4^, ^5^

Small skin grafts are widely used as a skin graft therapy in healing chronic wounds such as diabetic wounds. In this process, small sheets of the skin graft are laid over the cutaneous wound.^6^ Unfortunately, small skin grafts have limitations, like failures to neovascularize and ischemia–reperfusion injury.^7^ Hence, reconstructive surgeons are confronted with a significant challenge in improving the effectiveness of small skin grafting.

Recent years have witnessed the advent of chemically modified mRNA (modRNA) for expressing virtually any protein without triggering the innate immune responses both in vitro and in vivo.^8^ Several studies have demonstrated modRNA to highlight a new approach in treating ischemic disease.^9^, ^10^ A previous report has demonstrated that injecting SDF‐1α modRNA‐treated fibroblasts into the random skin flaps significantly improves the in vivo survival areas of the transplanted tissue by activating the SDF‐1α/CXCR4 axis pathway.^11^ Although the cellular delivery of modRNA has been found to enhance protein expression and induce potent angiogenesis, the transfection process is relatively complicated, restricting the clinical application in the future. However, it is still unknown and quite intriguing whether the SDF‐1α modRNA can be directly transfected into the skin tissues rather than being transfected into the cells.

Here, the small skin has been used as a ‘proof of concept’ vehicle for delivering and expressing the SDF‐1α modRNA into the full‐thickness skin defects in diabetic rats. We reported that, compared to the small skin alone, the small skin combined with chemically modified SDF‐1α mRNA delivery can enhance wound healing more efficiently and thus can be an alternative advanced strategy for treating diabetic wounds.

## 2 MATERIALS AND METHODS

### 2.1 Fabrication of small skin

The small skin was prepared according to the previous study,^12^ with minor modification. The neonate rats (SD, 3–5 days old) weighing approximately 20 g were used in this experiment. Firstly, they were intraperitoneally injected with sodium pentobarbital and then carefully rubbed with 75% ethyl alcohol over their backs before saline was washed. Then, a piece of full‐thickness skin (2 × 2 cm) was removed from the rat and cut into 5 × 5 mm with a scissor. Following this, the wound was rinsed thrice with phosphate‐buffered saline (PBS) after the skin debris was rinsed out. To eliminate the dermis and epidermis from the skin debris, threefold volumes of dispase were added and incubated overnight at 4°C. The epidermis (small skin) was washed with PBS and used for further experiments.

### 2.2 Animals model

**FIGURE 1:**
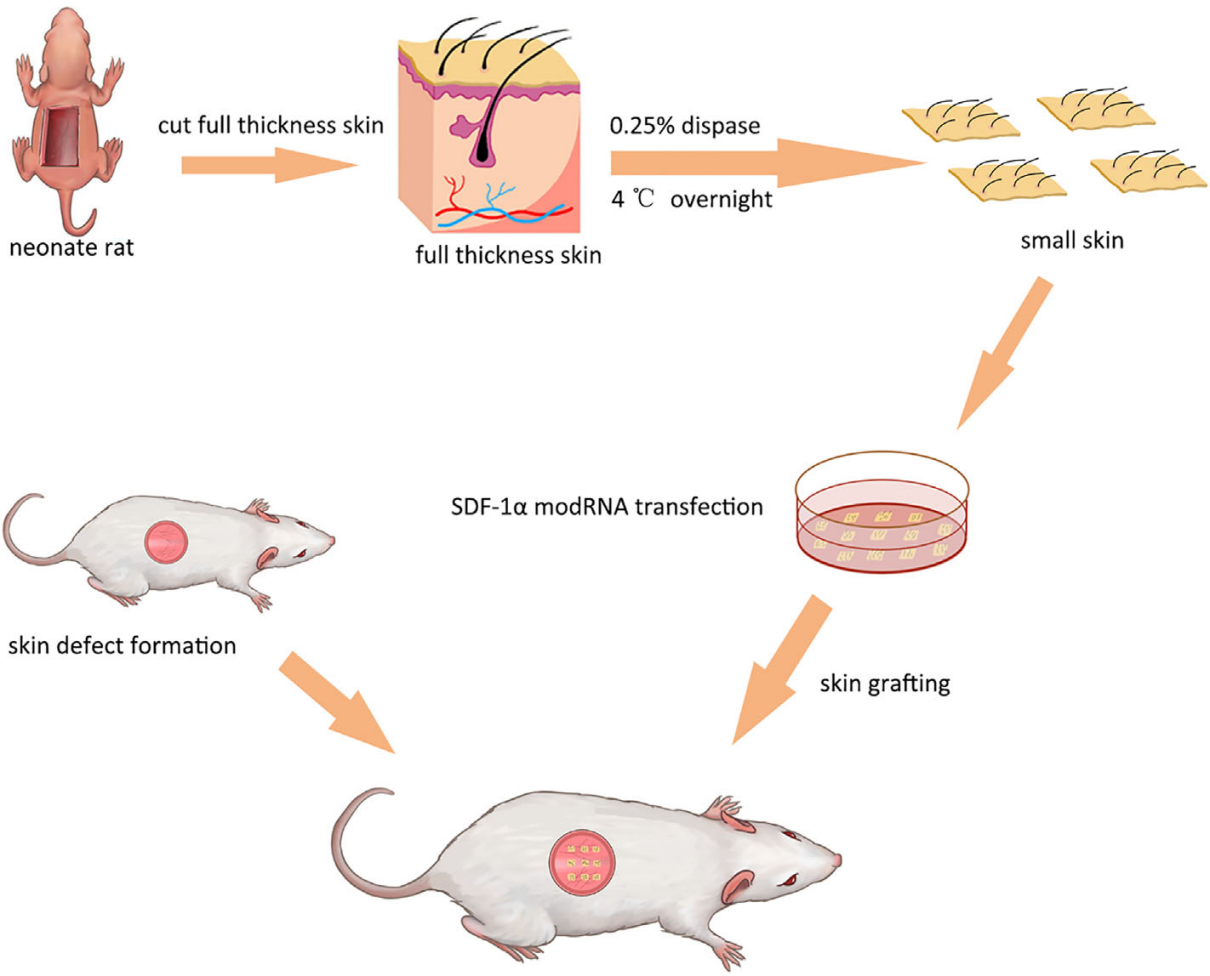
An illustration of how the small skin was transfected with the SDF‐1α modRNA and the design of the in vivo experiments

Rats (250–300 g) of the Sprague–Dawley (SD) species were obtained from the Chinese Academy of Sciences' Animal Center. The wounding operation was performed 5 weeks after injecting streptozotocin (STZ) into the rats. Following previous methods, the rats received an intraperitoneal injection of freshly prepared STZ (Sigma‐Aldrich) solution at a dose of 50 mg/kg body weight.^13^, ^14^ A dorsal skin wounding defect of a round shape with a diameter of 2 cm was made by excising the entire skin below the level of the dorsal fascia as the wounding defect model. An image of the wounds was captured immediately using a digital camera (Figure 1). According to the local treatment used for each rat, the rats were randomized into the control (Con group), small skin (MS group), luciferase modRNA transfected with the small skin (Luc + MS group), and SDF‐1α modRNA transfected with small skin (SDF + MS group). The group of MS 6, Luc + MS 6, and SDF + MS 6 were transplanted as six slices of autologous small skin, the group of MS 9, Luc + MS 9, and SDF + MS 9 were transplanted as nine slices of autologous small skin. The wounded area was measured by tracing the wound margins and evaluating as a percent area of the original wound using the ImageJ software (NIH). Each rat was provided with water and food in its cage. The study was approved by the Wenzhou Medical University's Animal Care and Use Committee (ethics code: wydw2017‐0159).

### 2.3 ModRNA synthesis and formulation

The mRNA was produced in vitro according to the previously described,^15^ transcribed using the RNA polymerase T7 on a DNA linearization template with generic 5′‐and 3′‐UTRs and poly‐A tails. The RNA was purified using the Ambion MEGA clear spin columns. Then, Antarctic Phosphatase (New England Biolabs) was introduced for 30 min at 37°C to remove the residual 5′‐phosphates upon initiation of treatment. Spectrophotometers from Thermo Scientific were used to quantify the purity of RNA as well as its concentration. The RNA was purified and resuspended at 1 μg/μl in 10 mM Tris HCl, 1 mM EDTA before use. The N1‐methylpseudouridine replaced uridine in mRNA. GFP and luciferase sequences were used as previously described.^16^ The sequence of SDF‐1α modRNA open reading frames is as follows:

ATGAACGCCAAGGTCGTGGTCGTGCTGGTCCTCGTG; CTGACCGCGCTCTGCCTCAGCGACGGGAAGCCCGTC; AGCCTGAGCTACAGATGCCCATGCCGATTCTTCGAA; AGCCATGTTGCCAGAGCCAACGTCAAGCATCTCAAA; ATTCTCAACACTCCAAACTGTGCCCTTCAGATTGTAG; CCCGGCTGAAGAACAACAACAGACAAGTGTGCATTG; ACCCGAAGCTAAAGTGGATTCAGGAGTACCTGGAGA; AAGCTTTAAACAAGTAA

### 2.4 Small skin transfection in vitro

According to a previously described method, the Lipofectamine Messenger MAXTM Reagent (Invitrogen, Life Technologies) was used to transfect small skins.^11^ In this study, four different transfection methods were designed according to the different amounts of modRNA added as follows.

#### 2.4.1 Method 1: 2 μl modRNA transfection system

Tube A: 2 μl modRNA (1 μg/μl) + 98 μl Opti‐MEM, incubated for 5 min.

Tube B: 5 μl Lipofectamine +95 μl Opti‐MEM, incubated for 5 min.

Then, mixtures of Tubes A and B were incubated at room temperature for 20 min.

#### 2.4.2 Method 2: 4 μl modRNA transfection system

Tube A: 4 μl modRNA (1 μg/μl) + 96 μl Opti‐MEM, incubated for 5 min.

Tube B: 10 μl Lipofectamine +90 μl Opti‐MEM, incubated for 5 min.

Then, mixtures of Tubes A and B were incubated at room temperature for 20 min.

#### 2.4.3 Method 3: 6 μl modRNA transfection system

Tube A: 6 μl modRNA (1 μg/μl) + 94 μl Opti‐MEM, incubated for 5 min.

Tube B: 15 μl Lipofectamine +85 μl Opti‐MEM, incubated for 5 min.

Then, mixtures of Tubes A and B were incubated at room temperature for 20 min.

#### 2.4.4 Method 4: 8 μl modRNA transfection system

Tube A: 8 μl modRNA (1 μg/μl) + 92 μl Opti‐MEM, incubated for 5 min.

Tube B: 20 μl Lipofectamine +80 μl Opti‐MEM, incubated for 5 min.

Then, the mixtures of Tubes A and B were incubated at room temperature for 20 min.

The cultured small skin samples were cultivated in 96‐well plates containing humidified atmospheres with 5% CO2 at 37°C in a different medium. The GFP modRNA expression in small skin was detected by photographing the small skin at 2, 4, 8, 12, 24, 48, and 72 h after transfection to seek appropriate modRNA transfection dose and time. Each well had 200 μl of Opti‐MEM added for the MS group. Each well of the Luc + MS group was filled with the same amount of mixture containing modRNA (2, 4, 6, and 8 μg). Similarly, the SDF‐1α modRNA was added equally to each well in the SDF + MS group.

### 2.5 Enzyme‐linked immunosorbent assay (ELISA)

As directed by the manufacturer, the ELISA kits (R&D Systems) were used. Small skin culture supernatants were collected in vitro at various timepoints (2, 8, 12, 24, 48, 72, 96, 120, and 144 h) after transfection and the levels of SDF‐1α were analysed by ELISA.

### 2.6 Histology and immunofluorescence

The paraffin‐embedded skin specimens were sectioned transversally after being embedded in paraffin (5 μm thick). The representative sections were stained for H&E according to standard procedures. Following the manufacturer's instructions, Masson's trichrome staining was performed to assess the fibrotic accumulation. The CD31and Ki67 were chosen to perform immunofluorescence to evaluate neovascularization and proliferation following conventional protocols. Briefly, following deparaffinization and rehydration, the sections were incubated with trypsin for 30 min at 37°C to retrieve antigens. The primary antibodies were applied separately overnight to the sections after blocking. Then, the specimens were incubated in the antibody dilution buffer for 2 h at room temperature with a secondary antibody. Finally, the section was then treated with diamino acrylamide and DAPI after rinsing it thoroughly with PBS. The cellular apoptosis assays were used to detect programmed cell death using the TUNEL Cell Apoptosis Detection Kit provided by Roche Applied Science according to the manufacturer's instructions.

### 2.7 Statistical analysis

The statistical analysis was carried out using the SPSS 20.0 software. An analysis of variance along with Tukey's test was used to compare the data from the control and treatment groups, while the Kruskal–Wallis *H* tests were used to compare the non‐parametric results. The data were presented as the mean ± standard deviation (SD). A *p* < 0.05 was considered statistically significant.

## 3 RESULTS

### 3.1 Optimization of the transfected modified SDF‐1α mRNA in small skin with protein expression in vitro

**FIGURE 2:**
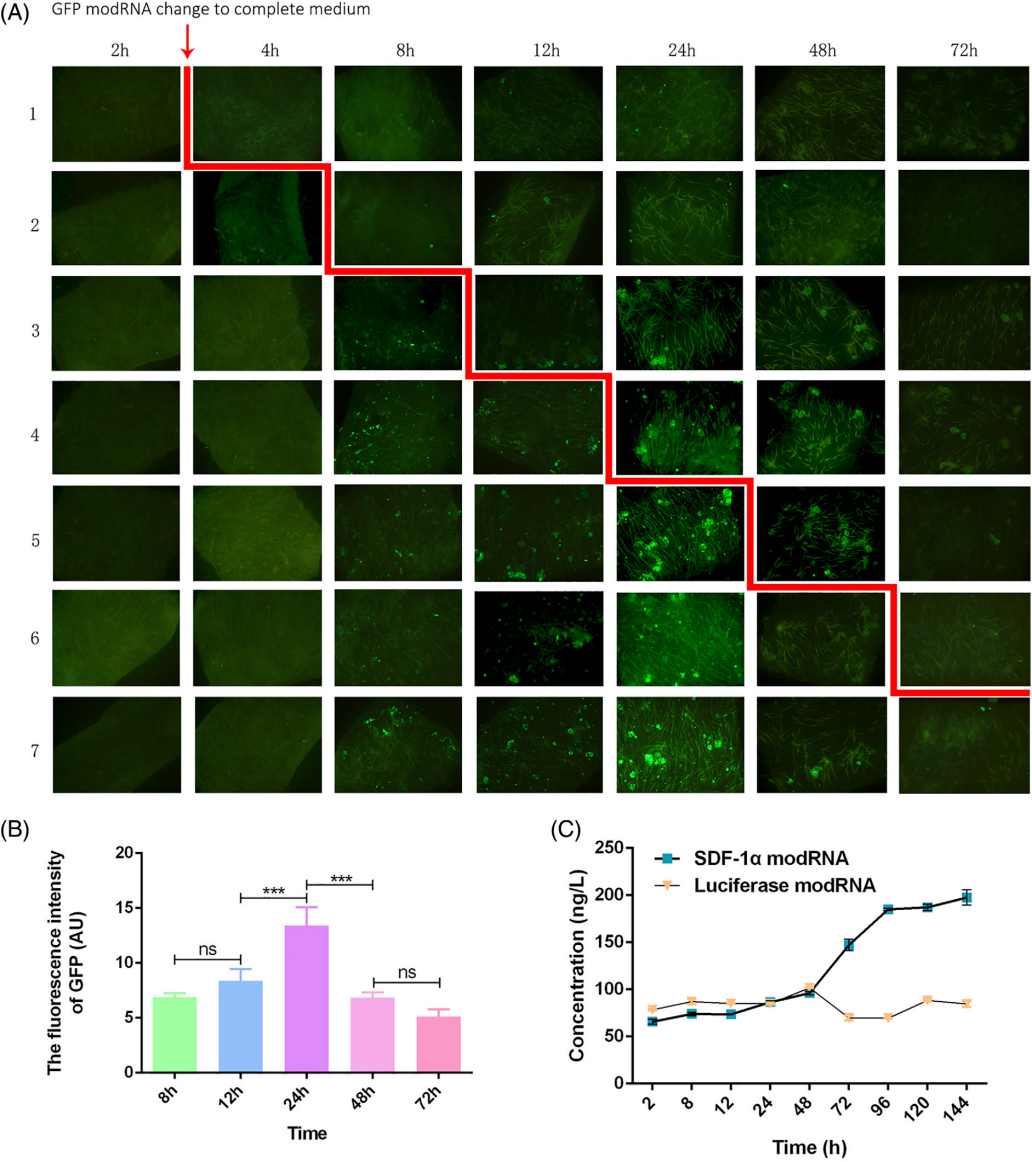
The small skin was transfected with modRNA. Fluorescence analysis of the expression of GFP observed in small skin by Method 3 and quantified in each group using the ImageJ software (A, B). The cumulative SDF‐1α concentration in small skin cells after transfection with modRNA for SDF‐1α (C)

To optimize the expression and transfection efficiency of the chemically modified mRNAs in small skin, four different transfection systems were evaluated based on the previous studies.^11^, ^17^ A reporter was constructed using GFP, when the GFP modRNA content went from 2 to 6 μl, a significant increase in the transfection efficiency in small skin cells was found. The GFP protein signals at each time point were increased significantly through the use of modified GFP mRNA, possibly due to a higher intake of mRNA and translation of the GFP proteins. However, when the content of GFP modRNA was increased to 8 μl, the result showed that the GFP modRNA decreased the transfection efficiency and observed a litter mean intensity signals of the GFP protein when compared to 6 μl of GFP modRNA (Figures 2A and S1–S3). To further ensure the optional transfection time, the effects of GFP transfection at different periods were examined and observed under a fluorescence microscope. The 6 μl modRNA transfection system was found to possess a transfection effect with the extension of the transfection time in the first 24 h and reached the peak at 24 h, and then the fluorescence expression of GFP gradually decreased with time (Figure 2B). The result of this study was consistent with that of the previous experiment.^11^ To detect the secreted SDF‐1α protein after transfection with SDF‐1α modRNA in Method 3, the ELISA was conducted immediately following transfection to determine the amount of SDF‐1α protein. In the luciferase modRNA‐transfected skin, the SDF‐1α expression was observed at a basic level, while the SDF‐1α concentration was significantly higher after 48 h of transfection with the SDF‐1α modRNA (Figure 2C). The results indicated that after transfection of the skin samples with the SDF‐1α modRNA, the SDF‐1α protein was the most highly expressed.

### 3.2 Small skin combined with the SDF‐1α modRNA enhanced the wound healing in full‐thickness skin defect rat model

**FIGURE 3:**
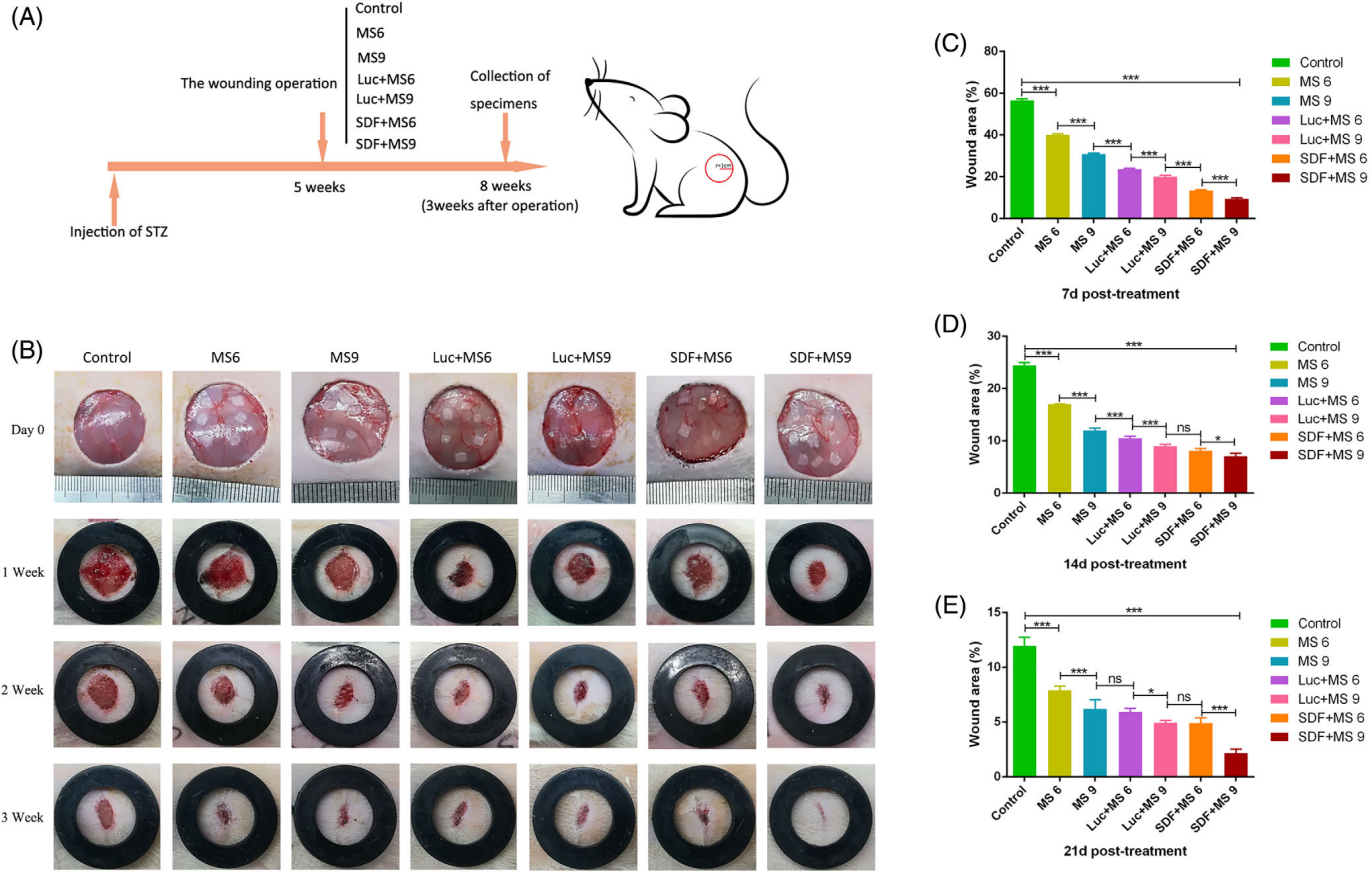
SDF‐1α modRNA transfected into the small skin promoted the full‐thickness wound defect healing in diabetics in vivo. The rats were randomized into control, MS 6, MS9, Luc + MS 6, Luc + MS 9, SDF + MS 6, and SDF + MS 9 groups (A). A quantitative evaluation of the wound area at Days 0, 7, 14, and 21 post‐treatment was done as well as a photograph of the wound area (B–E).

**FIGURE 4:**
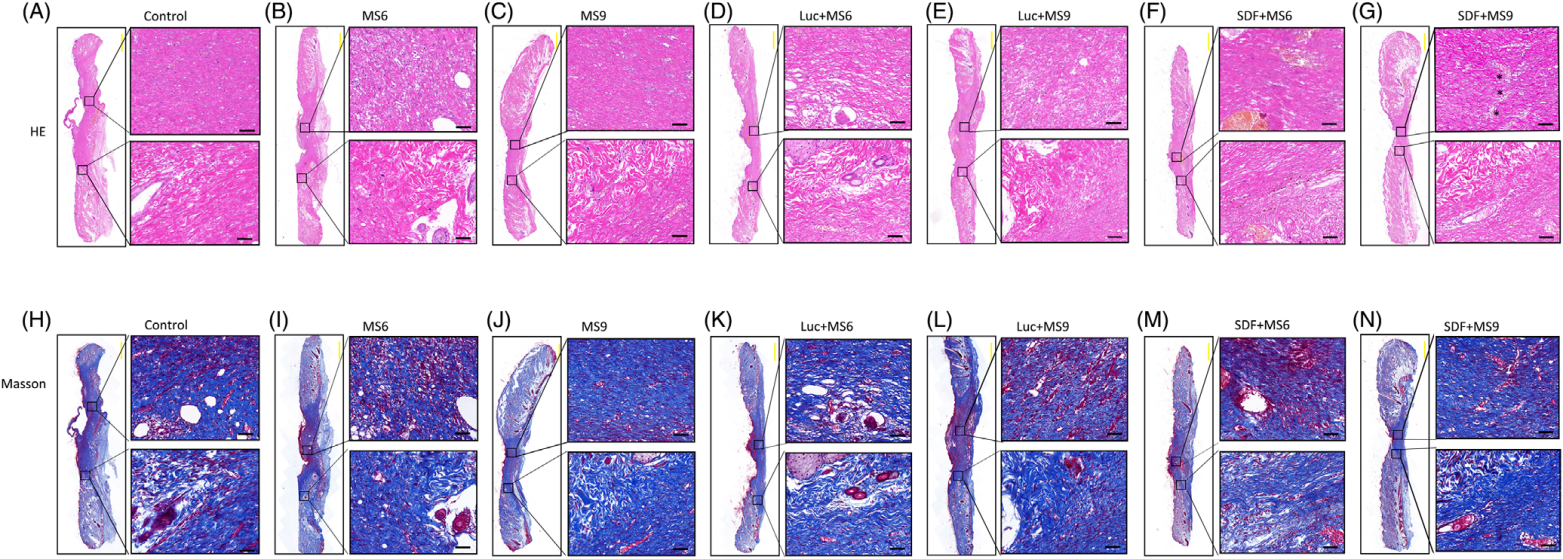
Histological evaluation of wound healing. Haematoxylin and eosin‐stained sections of tissue after 21 days of treatment in each group (A–N). Boxes with black dots indicate the locations of the microvessels (yellow scale bar: 1000 μm, black scale bar: 50 μm).

The original purpose was to investigate the effect of incorporating SDF‐1α modRNA into small skin on the healing of a full‐thickness skin defect in rats. The blood glucose level of the diabetic group was found to increase compared to that of the control group (Figure S4). The full‐thickness skin wounds (2 cm diameter) were made on the dorsal skin of each rat and the open wound area was photographed immediately (Figure 3A). Subsequently, the wound area was treated with different types of treatments and the wound area was observed by capturing photos during the transplantation process and analysed using ImageJ. There were no signs of infection like the presence of exudates or purulent drainage in any of the groups. Compared to all other groups, the wound area for the SDF + MS 9 group was smaller at Day 7 post‐treatment (Figure 3B,C). There was no statistical difference in the size of the wound area between the Luc + MS 9 and SDF + MS 6 groups, and the wound area of the SDF + MS 9 group was smaller than the SDF + MS 6 groups 14 days after treatment (Figure 3D). About 21 days after the treatment, in the SDF + MS 9 group, almost all of the wounded areas showed healing, whereas the other groups, specifically the control group, remained non‐healed (Figure 3E). It demonstrated that treating the small skin combined with the SDF‐1 α modRNA could enhance the healing of the full‐thickness skin defect wounds in the diabetic rat model. In addition, the SDF + MS 9 group exhibited a difference in the expression from the others in H&E staining. Moreover, the SDF + MS 9 group wound beds exhibited neoepidermis formation and marked vascularization at Day 21 post‐surgery (Figure 4A–G). Besides, according to Masson's trichrome staining, the collagen fibres appeared densely packed and densely stained in the SDF + MS groups, especially in the SDF + MS 9, while the collagen fibres appeared irregularly arranged and loosely packed in other groups (Figure 4H–N).

### 3.3 Small skin conditioned with the SDF‐1α modRNA promoted blood perfusion and inhibited apoptosis

**FIGURE 5:**
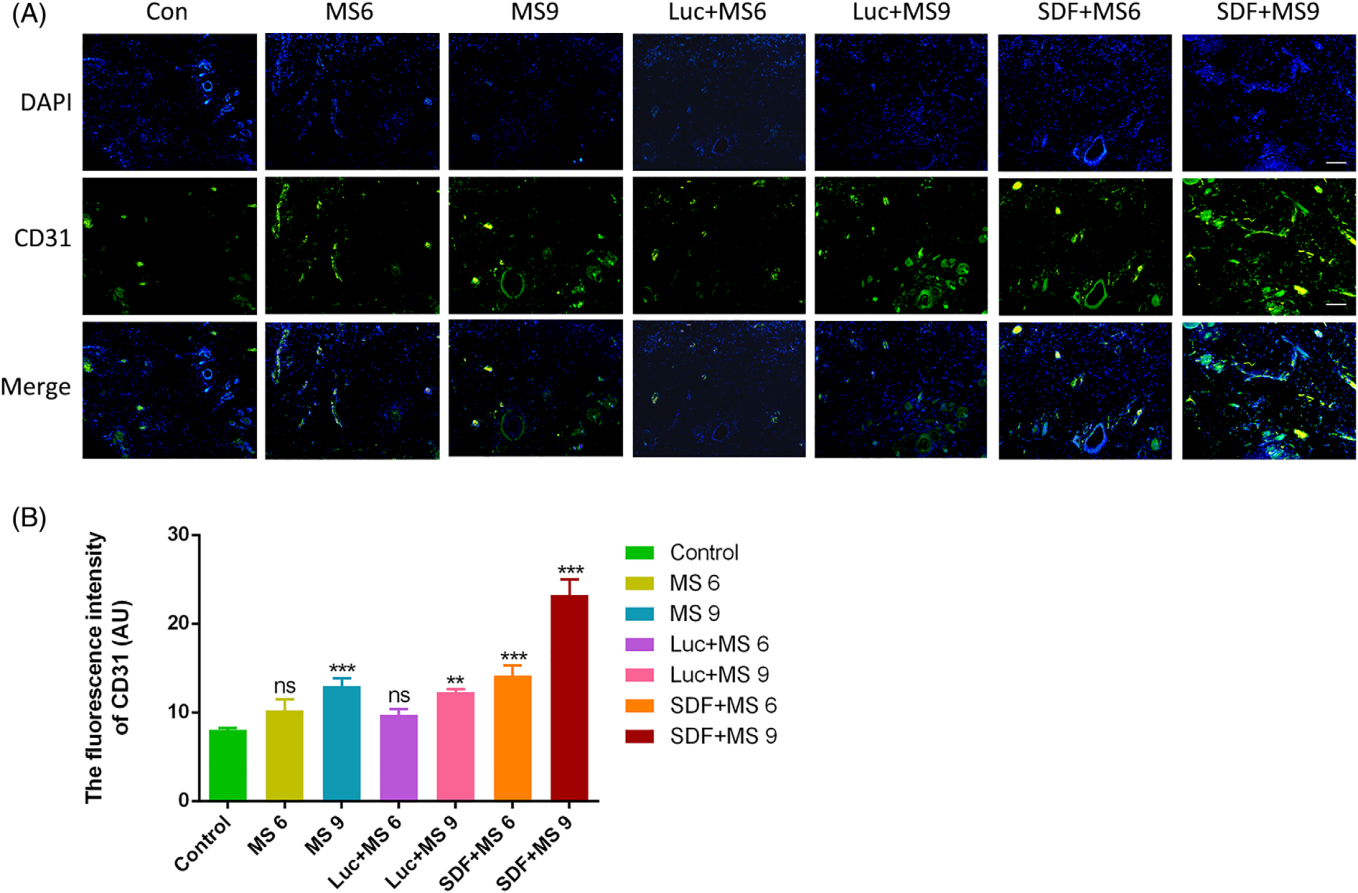
Neovascularization was promoted in wound healing by SDF‐1α modRNA treatment. The skin in each group was immunostained with fluorescence conjugated antibodies for anti‐CD31 antibodies and DAPI (A). (B) Analysing immunohistochemistry data (scale bar: 200 μm). Data represent the mean ± SD (*n* = 4). **p = 0.01–0.05, ***p < 0.01 versus control

**FIGURE 6:**
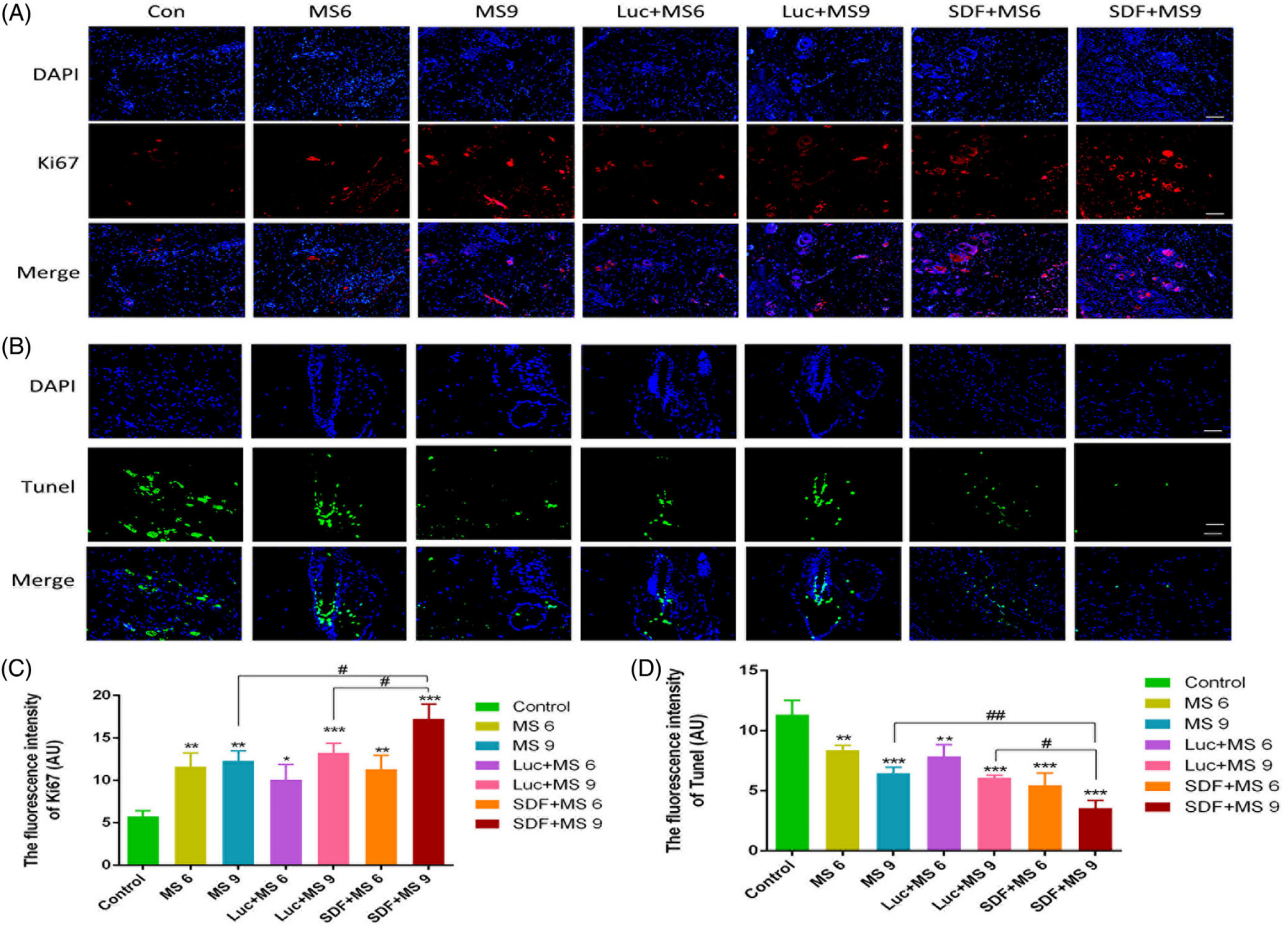
The proliferation of the wound healing cells was accelerated with the SDF‐1α modRNA treatment. The skin in each group was subjected to immunofluorescence staining with the anti‐Ki 67 antibodies and DAPI in each group (A). The TUNEL staining was used to determine the cell apoptosis (B). The immunohistofluorescence measurements were quantified (C, D). Data represent the mean ± SD (*n* = 4). * and **p = 0.01–0.05, ***p < 0.01 versus control. # and ##*p* = 0.01–0.05; scale bar: 200 μm

A subsequent experiment assessed the efficacy of the skin transfected with SDF‐1α modRNA in promoting wound healing. The CD31 immunostaining was used to determine the angiogenesis status of the wounds as the wound healing required neo‐vascularization (Figure 5A). The DAPI and CD31 staining strongly indicated that the SDF‐1α treatment significantly increased the number of newly formed vessels, suggesting that SDF‐1α assisted the wound healing and boosted angiogenesis. Quantitatively, the fluorescence intensity of the SDF‐1α treatment groups was found to be higher than that of the control, and the fluorescence intensity of MS 9 and Luc + MS 9 groups was significantly higher than that in the control group. However, there was no statistical difference in the fluorescence intensity between the MS6 and the control groups, nor was there any difference between the Luc + MS6 and the control groups (Figure 5B). Additionally, the immunofluorescent analysis demonstrated that the SDF‐1α modRNA‐transfected skin showed increased proliferation at Day 21 post‐surgery compared to that in the control groups, while the apoptosis was induced in the control groups (Figure 6A–D). Based on these results, the SDF‐1α transfected small skin‐treated fibroblasts‐secreted SDF‐1α effectively, indicating a critical role of these cells in wound healing.

## 4 DISCUSSION

The reconstruction of the vast areas of damaged skin, including wound contraction, delayed vascularization, and scar formation requires addressing several factors.^18^, ^19^ Skin grafting remains the ‘gold‐standard’ therapeutic option for treating massive area skin damage clinically.^20^ Nonetheless, following skin grafting a poorly vascularized wound bed can delay the wound healing.^21^ In a previous study, combining modRNA with cell therapy and tissue engineering was found to be effective for achieving tissue regeneration.^11^, ^17^ However, it is still unknown as to whether the modRNA can be directly transfected into tissues rather than transfected into the cells using in tissue regeneration.

The SDF‐1α modRNA was presumed to be able to translate into skin tissue to enhance wound healing in co‐action with the small skin. It was conventionally difficult to transfect the skin tissue with the modRNA due to the physical barrier of the skin mainly comprising the stratum corneum and tight junctions. Therefore, a method has been developed to detach the dermis and the epidermis performed as described by Zhang et al.^12^ To properly evaluate the efficiency of the SDF‐1α modRNA transfected into the small skin, the GFP modRNA was first used to validate the transfection of the small skin. The small skin was transfected with GFP modRNA referring to the transfection method of cells in our previous study.^11^ Unfortunately, the GFP fluorescence was not detectable at the small skin by Method 1 even when the transfection time was prolonged to 72 h (S1). Thus, we attempted to enhance the transfection effect by increasing the content of the GFP modRNA. When the GFP modRNA content increased to 6 μl, a strong GFP fluorescence signal was observed under a fluorescence microscope 24 h after transfection. However, the fluorescence intensity of GFP did not increase or even attenuated when the transfection time was extended and the content of the GFP modRNA increased (Figure S2,S3). The result was consistent with the previous studies demonstrating that modRNA was pulsed‐expressed proteins after transfection. Therefore, we used the same method (Method 3) to detect the change in the SDF‐1α content in the medium after being transfected with small skin by ELISA. Transfection of the SDF‐1α with modRNA resulted in higher SDF‐1α concentration after 48 h, while the GFP fluorescence was detected after 24 h. We suspect that it might take some time for SDF‐1α to be released from the cell to the medium due to the collagen structure being unique to the skin tissue.

As previously reported, SDF‐1 can enhance the effect of cytokines on the normal myeloid progenitors, and SDF‐1/CXCR4 signalling plays a crucial role in epithelialization by stimulating the migration and proliferation of the epidermal stem cell.^22^, ^23^ SDF‐1 may be the sole endogenous ligand for CXCR4. Tissue regeneration is based on the proliferation of the cells and the formation of vessels, which is the function of CXCR4.^24^ Several factors, such as injury, stress, hypoxia, and damage to the vascular system, can lead to enhanced expression of CXCR4.^25^ Recent evidence demonstrates that CXCR4‐derived stem cells and precursor cells and other tissue markers were expressed in the bone marrow during wound repair and regeneration. The inflammatory cells were attracted to the SDF‐1/CXCR4 axis for wound healing and chemotaxis and are activated for wound repair^26^, ^27^ and cell proliferation for injured tissues,^28^, ^29^ as well as the synthesis of the collagen for tissue remodelling.^30^

In this work, we found that combining small skin and SDF‐1α modRNA enhanced the repairing of the skin defects with an efficient cutaneous wound healing, compared to transplantation of the small skin or luciferase modRNA only. Moreover, the SDF + MS 6 group and Luc + MS 9 group did not show statistically significant wound area at 14 and 21 days. It is worth noting that the wound area decreased in the SDF + MS group compared to the Luc + MS group, suggesting that the SDF‐1α modRNA in combination with small skin can activate the SDF‐1/CXCR4 signal pathway, which might help to accelerate the healing of the skin wound. However, the dosing effect relationship between SDF‐1α content and wound healing needs further study. In line with these studies, this study reports that the pre‐treated fibroblasts with the SDF‐1α modRNA activated the SDF‐1/CXCR4 axis to enhance the random skin flap tissue regeneration.^11^

Meanwhile, during cell division, proliferation, growth, and apoptosis, apoptosis plays a crucial role in the cell. An important step in wound healing involves the elimination of the apoptotic cells.^31^, ^32^ Previous studies have reported that CXCR4 was related to cell apoptosis. The signalling pathway controlled by CXCR4 is a critical part of tissue regeneration for regulating apoptosis and moderating the biological environment.^33^ The SDF‐1/CXCR4 activation can modulate the antiapoptotic proteins via transcriptional SDF‐1α modRNA modes promoting the processes of wound repair and tissue regeneration (Figure 6).

## 5 CONCLUSION

This study has provided a descriptive report for identifying modRNA therapy as a potential therapeutic approach for treating full‐thickness skin defects. Importantly, the SDF‐1α modRNA was identified for the first time and could be effectively transfected into the skin tissue with the modRNA at appropriate concentrations and transfection time. Our study revealed that the combinatorial therapy of the modRNA with small skin may be promising for enhancing skin regeneration in the model diabetic tissues with full‐thickness damage.

## AUTHOR CONTRIBUTIONS

Conceptualization: Ke Liu, Wei Fu, and Jixin Xue. Methodology: Zucheng Luo, Yonghuan Song, and Li Shi. Software: Rui Zheng and Anyuan Wang. Validation: Zucheng Luo, Wei Fu, and Jixin Xue. Formal analysis: Haiting Xu, Huijing Wang, and Xiaoyan Li. Investigation: Zhenyu Tao and Huijing Wang. Resources: Jixin Xue. Data curation: Ke Liu. Writing—original draft preparation: Zucheng Luo. Writing—review and editing: Yujie Bian and Rui Zheng. Visualization: Yujie Bian and Wei Fu. Supervision: Jixin Xue. Project administration: Wei Fu and Jixin Xue. Funding acquisition: Jixin Xue. All authors have read and agreed to the published version of the manuscript.

## CONFLICT OF INTEREST

The authors declare no conflict of interest.

## Supporting information


**Figure S1.** Small skin transfected with modRNA. Fluorescence analysis of the expression of GFP observed in small skin by Method 1.
**Figure S2**. Small skin transfected with modRNA. Fluorescence analysis of the expression of GFP observed in small skin by Method 2.
**Figure S3**. Small skin transfected with modRNA. Fluorescence analysis of the expression of GFP observed in small skin by Method 4.
**Figure S4**. Blood glucose of rats in the control and diabetic groups.

## Data Availability

The original contributions presented in the study are included in the article/supplementary material. Further inquiries can be directed to the corresponding author(s).
