# Corrigendum

**DOI:** 10.1111/cpr.13421

**Published:** 2023-06-20

Correction to “Combination of chemically modified SDF‐1α mRNA and small skin improves wound healing in diabetic rats with full‐thickness skin defects”.

In Luo et al.,^1^ the following errors were published.

These are errors in Figure 5A in CD31 result in MS6 group and in Figure 6B in Tunel result in Con group.

**FIGURE 5:**
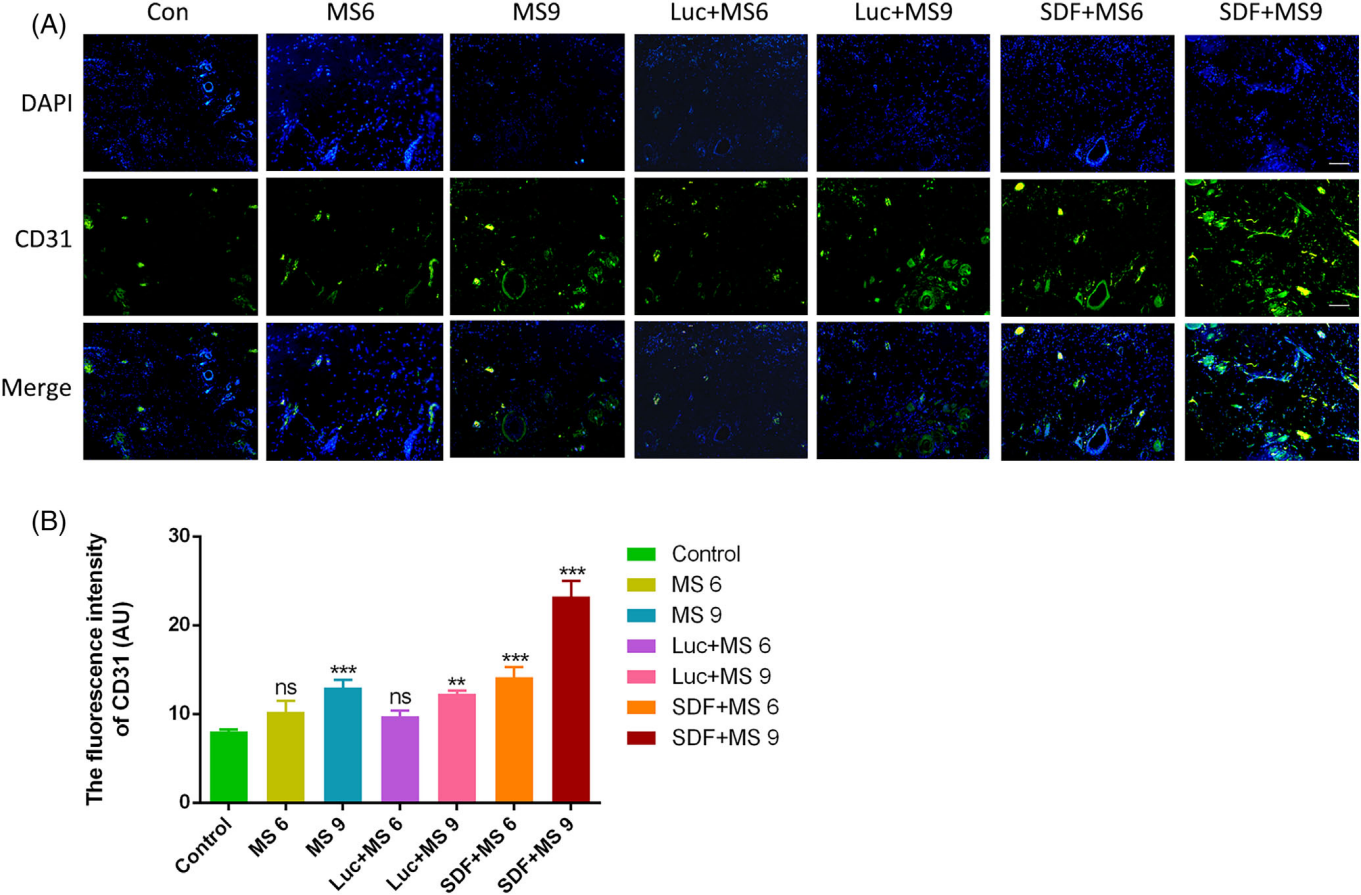


**FIGURE 6:**
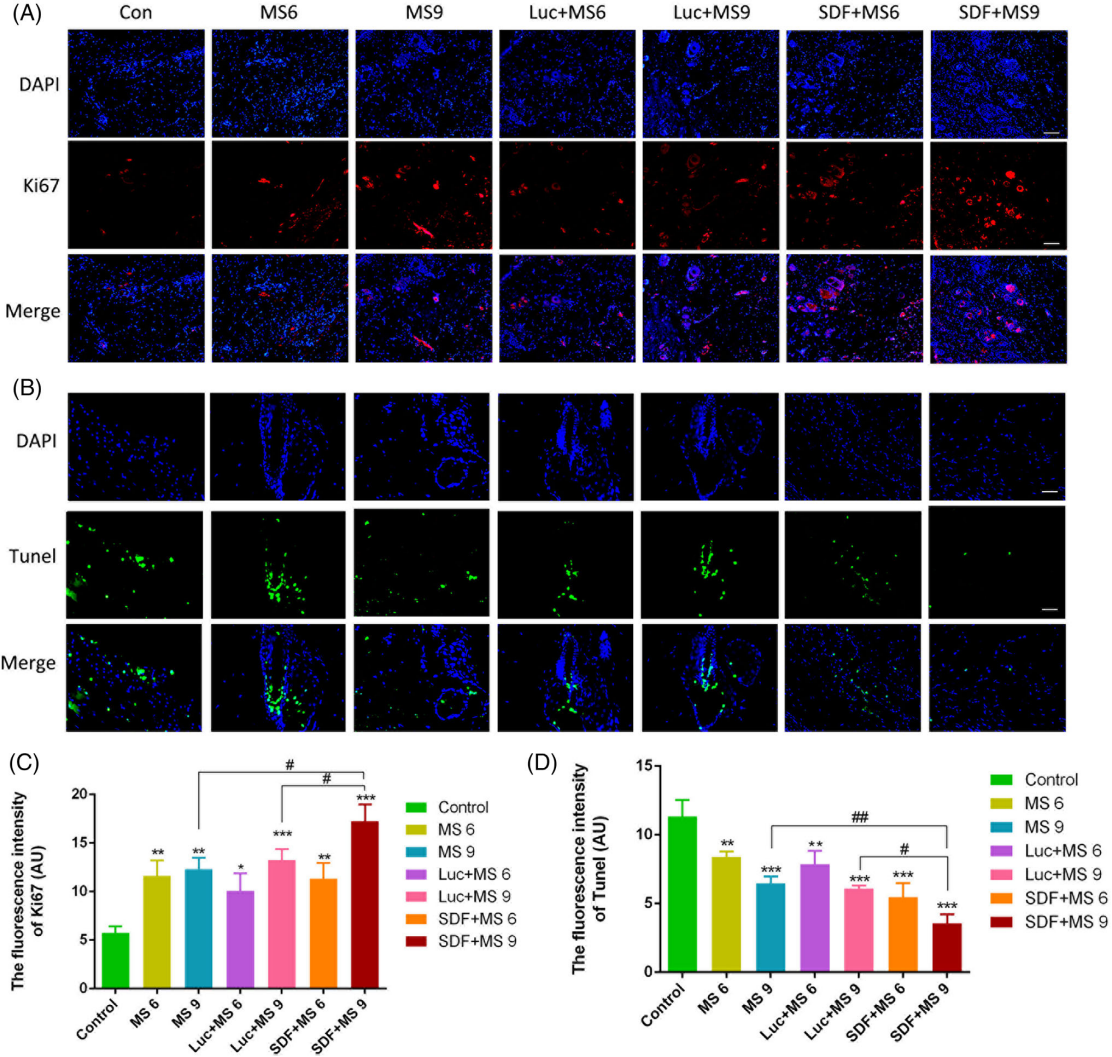


The corrected Figures 5 and 6 are provided below. The error does not affect the results and conclusion of this article. The authors apologize for any inconvenience caused.
