# Supplementary material for: Combination of chemically modified SDF‐1α mRNA and small skin improves wound healing in diabetic rats with full‐thickness skin defects
Source: Cell Prolif. 2022 Aug 6;55(12):e13318. doi: 10.1111/cpr.13318 (PMC9715359; doi:10.1111/cpr.13318)
Supplement: Supplementary file 1 — Figure S1. Small skin transfected with modRNA. Fluorescence analysis of the expression of GFP observed in small skin by Method 1. Figure S2. Small skin transfected with modRNA. Fluorescence analysis of the expression of GFP observed in small skin by Method 2. Figure S3. Small skin transfected with modRNA. Fluorescence analysis of the expression of GFP observed in small skin by Method 4. Figure S4. Blood glucose of rats in the control and diabetic groups. [file CPR-55-e13318-s001.docx]

**
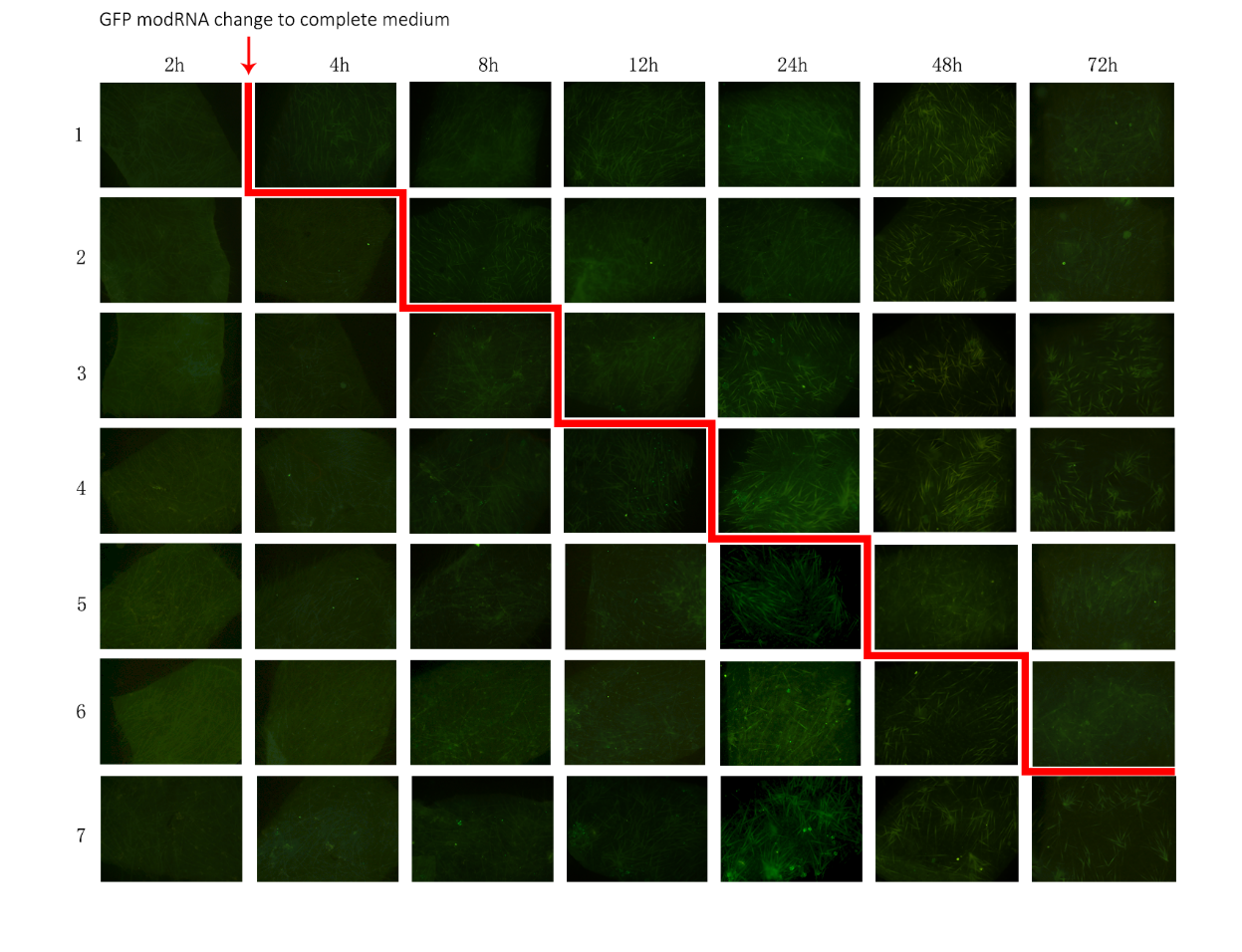
**

**Support date 1 (S1)**. Small skin transfected with modRNA. Fluorescence analysis of the expression of GFP observed in small skin by Method 1.


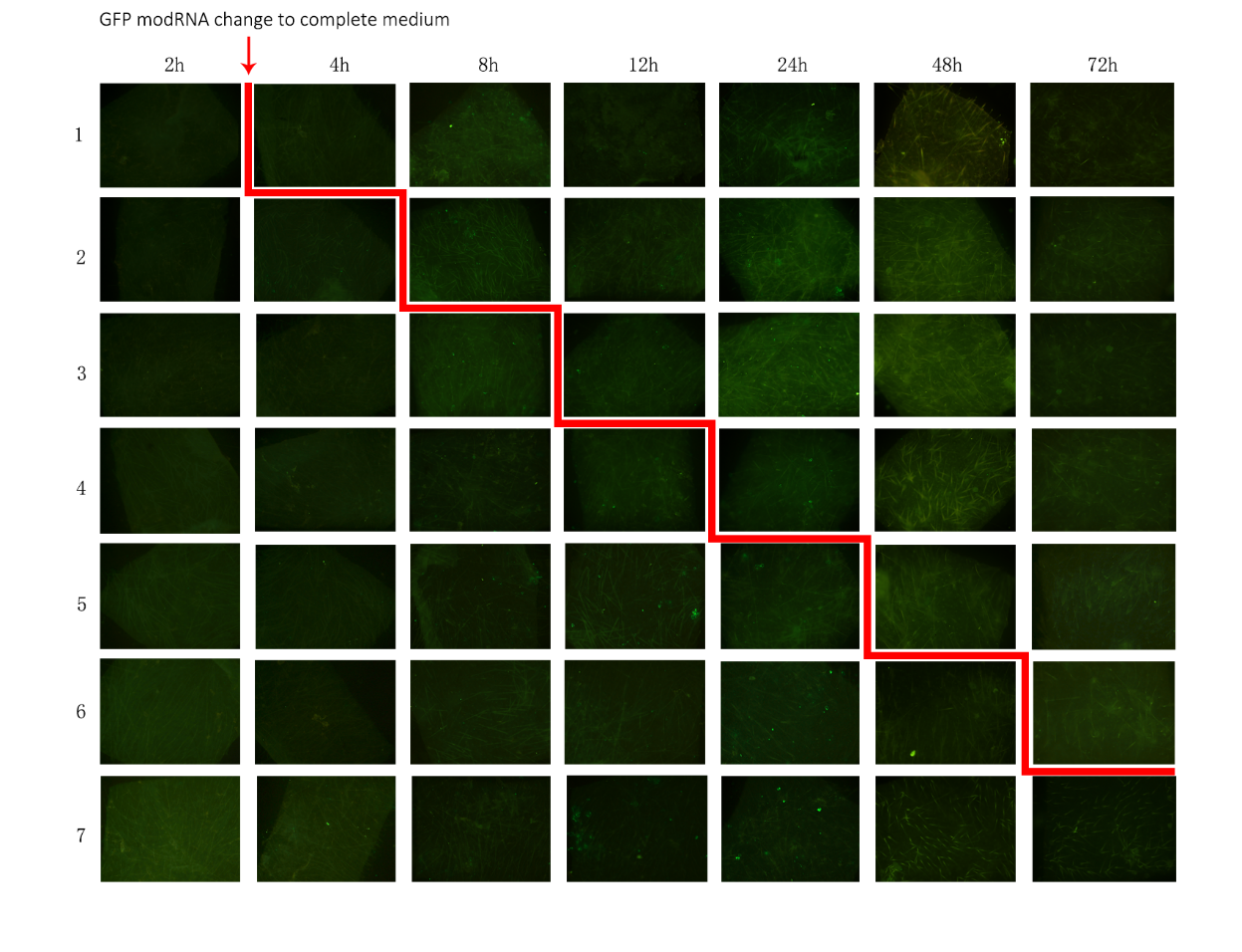


**Support date 2 (S2)**. Small skin transfected with modRNA. Fluorescence analysis of the expression of GFP observed in small skin by Method 2.


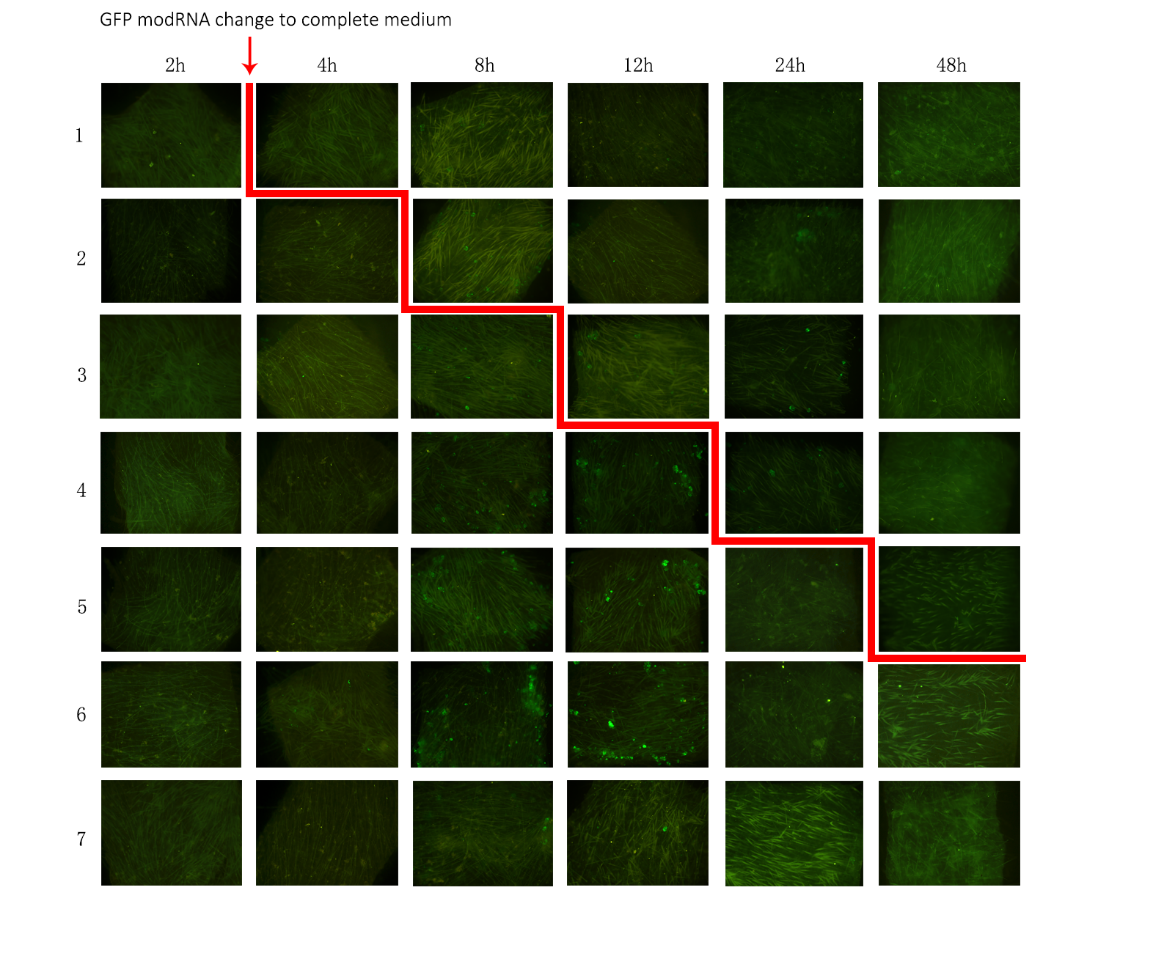


**Support date 3 (S3)**. Small skin transfected with modRNA. Fluorescence analysis of the expression of GFP observed in small skin by Method 4.


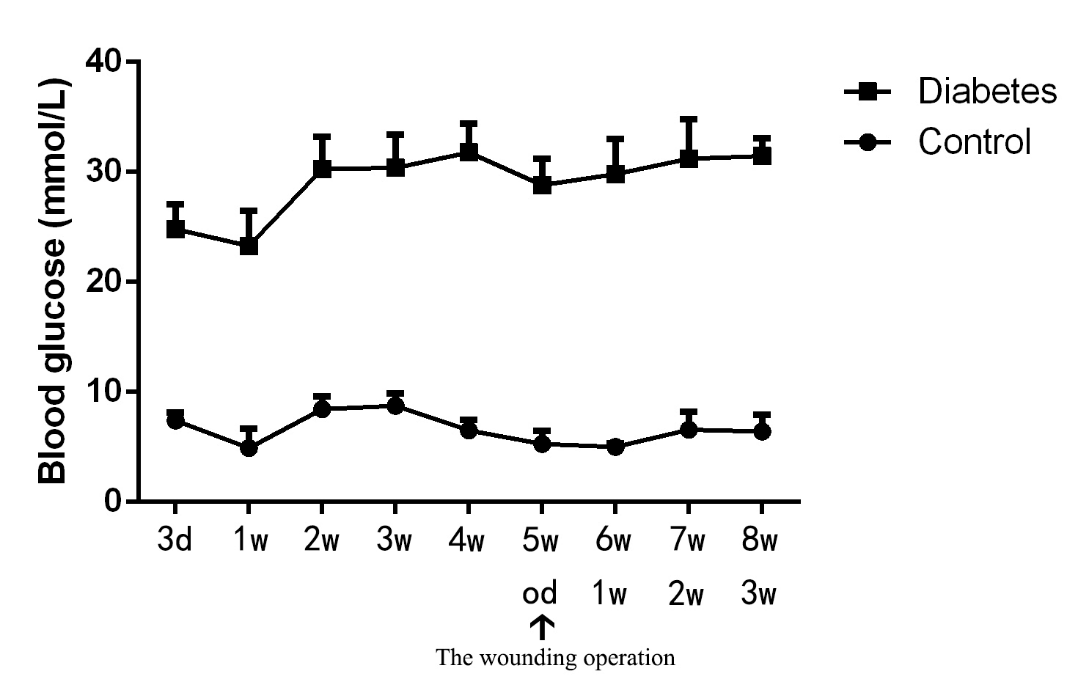


**Support date 4 (S4)**. Blood glucose of rats in the control and diabetic groups.
